# Meta-analysis of genomic variants in power and endurance sports to decode the impact of genomics on athletic performance and success

**DOI:** 10.1186/s40246-024-00621-9

**Published:** 2024-05-17

**Authors:** Aikaterini Psatha, Zeina N. Al-Mahayri, Christina Mitropoulou, George P. Patrinos

**Affiliations:** 1 Laboratory of Pharmacogenomics and Individualized Therapy, Department of Pharmacy, School of Health Sciences, University of Patras, University Campus, Rion, 265 04 Patras, Greece; 2 The Golden Helix Foundation, London, UK; 3 Clinical Bioinformatics Unit, Department of Pathology, Faculty of Medicine and Health Sciences, Erasmus University Medical Center, Rotterdam, The Netherlands; 4 Department of Genetics and Genomics, College of Medicine and Health Sciences, United Arab Emirates University, Al-Ain, Abu Dhabi, UAE; 5 Zayed Center for Health Sciences, United Arab Emirates University, Al-Ain, Abu Dhabi, UAE

**Keywords:** Athletic performance, Genomic variants, Meta-analysis, Endurance sports, Power sports, *ACE*, *ACTN3*

## Abstract

Association between genomic variants and athletic performance has seen a high degree of controversy, as there is often conflicting data as far as the association of genomic variants with endurance, speed and strength is concerned. Here, findings from a thorough meta-analysis from 4228 articles exploring the association of genomic variants with athletic performance in power and endurance sports are summarized, aiming to confirm or overrule the association of genetic variants with athletic performance of all types. From the 4228 articles, only 107 were eligible for further analysis, including 37 different genes. From these, there were 21 articles for the *ACE* gene, 29 articles for the *ACTN3* gene and 8 articles for both the *ACE* and *ACTN3* genes, including 54,382 subjects in total, from which 11,501 were endurance and power athletes and 42,881 control subjects. These data show that there is no statistically significant association between genomic variants and athletic performance either for endurance or power sports, underlying the fact that it is highly risky and even unethical to make such genetic testing services for athletic performance available to the general public. Overall, a strict regulatory monitoring should be exercised by health and other legislative authorities to protect the public from such services from an emerging discipline that still lacks the necessary scientific evidence and subsequent regulatory approval.

**Supplementary Information:**

The online version contains supplementary material available at 10.1186/s40246-024-00621-9.

## Introduction

The impact of genomic variants on sports performance, particularly in terms of endurance and power, is a highly debated subject within human genomics and sports science. Research has identified over 250 genomic variants across more than 140 genes linked to traits influencing athletic performance. However, only a select few of these variants are believed to play a decisive role in establishing the caliber of an elite athlete [10], suggesting that there are significant gaps in our understanding of the impact of genetic factors on athletic performance or various related phenotypes. It is estimated that the degree of the genetic contribution to athletic performance is approximately 50%, while endurance and power-related characteristics are within a range of 44–68% and 48–56%, respectively [6, 15]. On top of these, non-genetic factors, such as different training methods, diet, and ethnicity, are held accountable for the remaining variation of performance between athletes, while environmental factors have a strong impact on either modulating gene expression or epigenetic modifications [13]. As such, all these parameters play a pivotal role in determining the overall athletic performance, metaphorically a multi-piece jigsaw puzzle, making this virtually impossible to simulate and predict with the existing level of evidence.

Given the current state of evidence, it is both premature and fraught with risk to leverage human genomics knowledge to predict exercise and sports performance or enhance existing training methodologies [12]. This caution stems primarily from the considerable heterogeneity and conflicting findings in research efforts aimed at correlating the human variome with athletic prowess [7], similar to the situation of providing genetic-based advice for individualizing dietary choices [4, 8]. The heterogeneity and ambiguity observed in this field can be attributed to several key factors, including limitations in study design. These limitations mostly stem from imprecise phenotypic measurements and the often-vague classification of individuals as elite athletes, especially in sports where victories in World Championships and/or Olympic Games are determined in milliseconds. Furthermore, the typically small cohorts of athletes involved in these studies compound the issue, rendering attempts to correlate loosely defined phenotypes with genomic variants futile and rendering the results of such studies challenging to interpret. These shortcomings not only apply to single variants analysis but also, most importantly, to multi-variant and pathway analysis derived from genome-wide association studies. Notably, for several years and despite the existing lack of evidence, several private genetic laboratories have offered such genetic testing services for athletic performance to the general public, using the direct-to-consumer approach. This practice raises significant ethical concerns, particularly in terms of misleading the general public about the actual efficacy and value of these genetic testing services [7].

To date, there has yet to be a thorough meta-analysis that synthesizes the existing body of evidence regarding the association between genomic variants and athletic performance, encompassing diverse aspects such as endurance and power. This study aimed to provide a comprehensive evaluation of the influence of genetics on endurance and power-oriented sports. The goal was to elucidate the potential applicability of genomics in predicting sports talent and optimizing training methodologies with a high degree of clarity and precision.

## Methods

### Literature search

A thorough literature search was performed in the PubMed literature database and Google Scholar from 1945 until March 2024. There was no restriction on the publication date of the studies. A combination of the following keywords was used: “sport”, “athlete”, “elite”, “endurance”, “power”, “strength”, “speed”, “performance-enhancing gene polymorphisms (PEPs)”, “total genotype score”, “polymorphism”, “genetic”, “marker”, and “genetic test”.

More specifically, the following searches were performed in PubMed:

- “Sport AND genetic test AND endurance”,
- “Performance-enhancing gene polymorphisms (PEPs)”,
- “((sports [Title/Abstract]) AND (endurance [Title/Abstract])) AND (genetic [Title/Abstract])”,
- “(((sports [Title/Abstract]) AND ((power [Title/Abstract]) OR (strength [Title/Abstract])) AND (genetic [Title/Abstract])))”,
- “Total genotype score AND sport” on May 24, 2022, with filters “Free full text, Humans, English” and brought 10 results.

The following searches were performed in Google Scholar:

- “Sports" & (“power” OR “endurance” OR “strength” OR “speed”) & (“gene test” OR “genetic test”)”,
- “(Sport OR athlete OR elite) AND (endurance OR power OR strength) AND (marker OR polymorphism OR genetic)”,

In total, from the above searches, 4228 articles were retrieved (see below).

### Inclusion and exclusion criteria

Original articles published in English involving case–control and cohort studies were reviewed. The studies included investigated any genomic variant in elite athletes (study group; highly elite, elite, sub-elite, professional, etc*.*) compared to a control group (non-athlete) population. The studies had to include athletes of any nationality engaged in any sport requiring power or endurance skills. Classification of the athletes as highly elite, elite sub-elite, and average followed Druzhevskaya and coworkers’ [3] classification.

Studies were excluded from the downstream analysis based on the following criteria:

- Were published in a language other than English,
- Their full text was not available,
- Found more than once through the different searches carried out,
- Included non-human samples,
- Consisted of review articles, conference abstracts, commentaries, or other non-original studies,
- Were investigating intermediate-level athletes or athletes whose sport fell under the mixed category, meaning it required a combination of power and endurance skills,
- Did not include a control population,
- Met the inclusion criteria applied in the specific meta-analysis but did not provide the necessary genotype frequency data, and
- The frequency of the control population genotypes was incompatible with the expected frequency defined by the Hardy–Weinberg Equilibrium (HWE) principle.

From the original publications that were finally included in the study, the following data were extracted: (a) first author and year of publication, (b) distribution of genotypes for each of the genomic variants studied, between athletes and controls (non-athletes), (c) characteristics related to the study design and the population included (gender, ethnicity, number of athletes and controls), (d) information about the sport and whether it requires power or endurance skills, and (e) information on the genotypes frequency in both athletes and control groups.

### Statistical analysis

Odds ratios (ORs) with 95% confidence intervals (CIs) were calculated. Also, the *p*-value resulting from the χ^2^ test was determined to assess whether the study sample was in Hardy–Weinberg Equilibrium. 1-side and 2-side t-test was performed to assess the statistical significance of the associations between athletic performance and genomic variants, with a *p*-value of < 0.05 considered to be statistically significant.

## Results

### Literature search

**Fig. 1:**
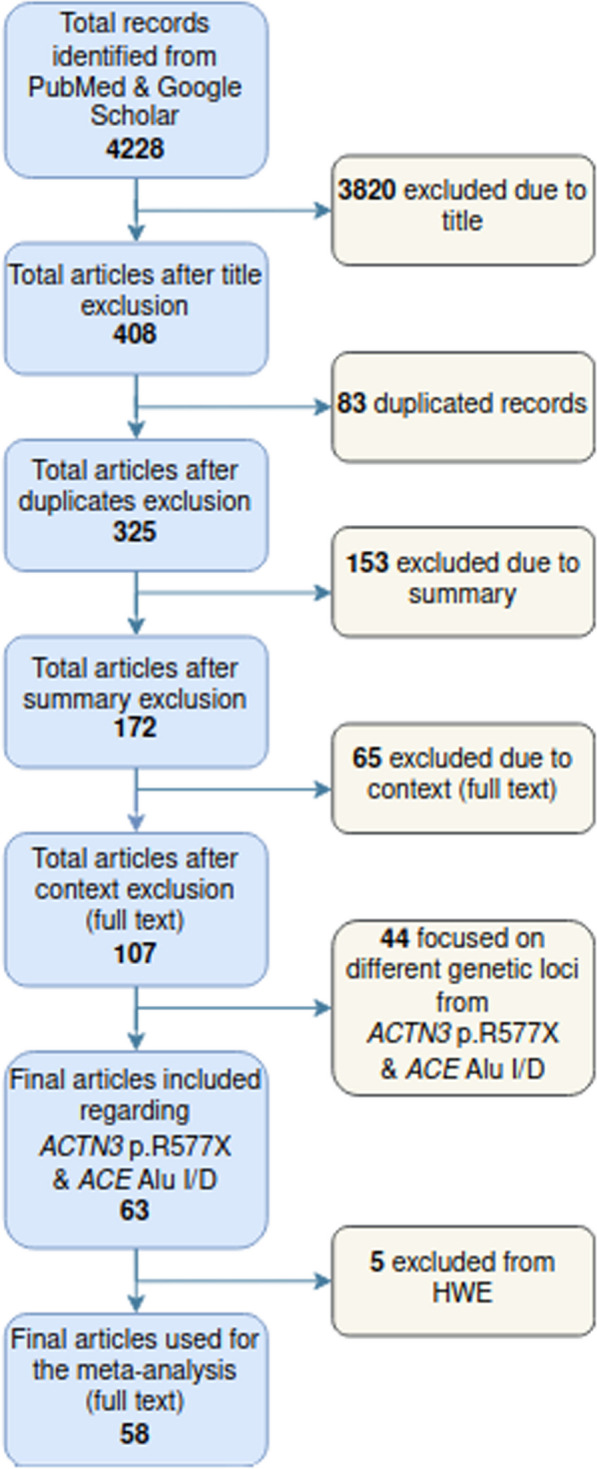
Flow chart showing the process for selecting the studies included in the meta-analysis (see also Supplementary Table 1)

Our initial literature search, with the keywords described above, revealed 4228 articles in total, from which 3820 were excluded based on the article title. From the 408 remaining articles, 83 duplicate entries were removed, together with 153 additional articles, based on the content of the abstract. From the 172 remaining articles, 65 additional articles have been excluded, from which 7 articles due to the lack of the full text, 29 articles due to unavailable datasets, 15 articles that did not include comparison with a control sample, and one article that included data from another study (flow chart provided in Fig. 1). The remaining 107 articles that were included in the analysis involved 57 genetic loci (Table 1). From the above, only 63 studies pertaining to the angiotensin-converting enzyme (*ACE*) Alu insertion/deletion (Alu I/D) and alpha actinin-3 gene (*ACTN3)* p.R577X variants, from which 5 were removed since the alleles were not in Hardy Weinberg equilibrium in the study sample. As such, 58 articles allowed for a proper meta-analysis, from which 21 articles for the *ACE* gene, 29 articles for the *ACTN3* gene and 8 articles for both the *ACE* and *ACTN3* genes, including a total of 11,501 (6215 endurance and 5286 power) athletes and 42,881 control subjects (Table 2).

**Table 1:** Outline of the genes/variants and the corresponding number of articles that have been identified within the scope of our literature search

Gene	Variant	No of articles
***ACE***	**Alu I/D**	**29**
	c.A22982G	2
***ΑCTN3***	**p.R577Χ**	**37**
	p.Q523R	1
*ADRA2*	c.1291C/G (rs1800544)	1
	c.1291A/G (rs553668)	1
*‍ADRB1*	rs1801252	1
*ADRB2*	c.46A/G / p.R16G (rs1042713)	2
	c.79C/G / p.Q27E (rs1042714)	2
	p.T164I	1
*ADRB3*	rs4994	1
*AGTR2*	c.G1675A/C	1
*AGT*	p.rs699	6
*AMPD1*	c.C34T	5
*BDKRB2*	rs5810761	1
*CKM (CKMM)*	c.*800A > G	2
	rs8111989	2
*CYP2D6*	c.775delA/p.R259Gfs	1
	c.506–1G/A	1
	*10 (rs1065852)	1
*FAAH‍*	rs324420	1
*FTO*	rs9939609	2
*GALNTL6*	rs558129	1
*GDF-8*	p.K135R	2
*GSTM1*	“Null” allele	1
*HIF1A*	p.P528S	4
*HFE*	p.H63D	1
	p.C282Y	1
*IGF*	c.-1245C/T (rs35767)	1
	c.275124A/C (rs1464430)	2
	c. rs680 AG	2
*IL6*	c.174G/C	2
*MB*	rs7293	1
*MCT1*	p.D490E (rs1049434)	2
*‍MYOZ2*	rs9995277	1
*‍MYOZ3*	rs116090320	1
*NOS3*	c.786T > C	2
	p.E298D	4
*NRF2*	rs12594956	4
	rs7181866	3
	rs8031031	3
*‍PPARA*	c.2528G > C (rs4253778)	9
*‍PPARD*	c.T294C (rs2016520)	4
	p.G482S (rs2267668)	1
	rs1053049	2
*‍PPARG*	c.2528G > C/p.P12A (rs1801282)	2
*PPARGC1A*	c.1444G > A/p.G482S (rs8192678)	9
*PPARGC1B‍*	p.A203P	1
*‍TFAM*	rs1937	1
	rs2306604	1
*UCP2*	c.866G/A/p.A55V	2
*UCP3*	c.55C/T	1
*VEGFR2*	c.1416A > T/p.Q472H	1
*VDR*	F/f (rs10735810)	1

The two gene / variant combinations that have been included in the downstream meta-analysis (*ACE* Alu I/D and *ACTN3* p.R577X) are indicated in boldface. Some of these articles address the correlation with more than one genes (see also “Methods” and Supplementary Table 1)

**Table 2:** Number of endurance and power athletes and controls that were included in the present meta-analysis for the *ACE* Alu I/D and *ACTN3* p.R577X variants, respectively

Variant	Endurance	Control	Power	Control
*ACE* Alu I/D	2504	7155	1635	9650
*ACTN3* p.R577X	3711	11,169	3651	14,907
Total	6215	18,324	5286	24,557

Endurance sports included in our analysis were rowing, skiing, skating, water polo, handball, duathlon, triathlon, pentathlon, medium- and long-distance running, swimming, and cycling. Power sports included in our analysis were wrestling, boxing, judo, bodybuilding, powerlifting, javelin throw, high jump, long jump, triple jump, football, kayak, volleyball, ice hockey, and short-distance running, swimming, and cycling.

### Comparisons

For the *ACE* I/D variant, we have performed the following comparisons: (a) *ACE* II + ID versus *ACE* DD, and (b) *ACE* II versus *ACE* ID + DD for the following case/control groups: (i) power athletes versus controls, (ii) endurance athletes versus controls, and (iii) power AND endurance athletes versus controls**.** For the *ACTN3* p.R557X variant, we have performed the following comparisons: (a) *ACTN3* p.557RR + p.557RX versus *ACTN3* p.557XX, and (b) *ACTN3* p.557RR versus *ACTN3* p.557RX + p.557XX for the following case/control groups: (i) power athletes versus controls, (ii) endurance athletes versus controls, and (iii) power AND endurance athletes versus controls. Our findings are summarized in Table 3.

**Table 3:** Assessing the association of the *ACE* II / ID / DD and *ACTN3* p.577RR, p.577RX and p.577XX genotypes by comparing their prevalence among endurance and/or power athletes and control subjects (see also Supplementary Figs. 1– 12)

Comparison (athletes vs control)	OR (95% CI)	*p*-value	Suppl Figures
*ACE* II + ID versus *ACE* DD			
Endurance athletes	1.351 (0.908–2.042)	0.915	1
Power athletes	0.825 (0.393–2.089)	0.491	2
Power AND endurance athletes	1.189 (0.728–1.946)	0.956	3
*ACE* II versus *ACE* ID + DD			
Endurance athletes	1.305 (0.827–2.091)	0.602	4
Power athletes	0.881 (0.458–1.620)	0.718	5
Power AND endurance athletes	1.222 (0.622–2.404)	0.996	6
*ACTN3* p.577RR + p.577RX versus *ACTN3* p.577XX			
Endurance athletes	1.210 (0.635–2.722)	0.852	7
Power athletes	1.672 (1.077–2.597)	0.995	8
Power AND endurance athletes	1.635 (0.928–2.939)	0.983	9
*ACTN3* p.577RR versus *ACTN3* p.577RX + p.577XX			
Endurance athletes	1.180 (0.889–1.612)	0.340	10
Power athletes	1.333 (0.599–3.158)	0.720	11
Power AND endurance athletes	1.167 (0.776–1.839)	0.945	12

In brief, the attempt to correlate the *ACE* Alu I/D variant with athletic performance in both power and endurance athletes compared to controls did not yield statistically significant results (Table 3, Supplementary Figs. 1–6). Similarly, there were no statistically significant differences in the prevalence of *ACE* Alu I and *ACE* Alu D alleles between athletes and control subjects (Supplementary Figs. 13,14).

Regarding the association of the *ACTN3* p.R577X allele with athletic performance, this did not yield any statistically significant results either (Table 3, Supplementary Figs. 7–12). At the same time, there were no statistically significant differences when comparing the prevalence of the *ACTN3* p.577R allele with the *ACTN3* p.577X allele in endurance and power athletes with control subjects (Supplementary Figs. 15, 16).

## Discussion

The current meta-analysis investigated the potential association between specific genomic variants and athletic performance. This study represents the most extensive meta-analysis conducted thus far to explore this pertinent issue, which holds significance not only within the realm of research but also from an ethical standpoint. Contrary to the few other systematic reviews and meta-analyses in the literature that only focused on the *ACE* and the *ACTN3* genes [5, 11], this study aimed to identify correlations between genetic loci and athletic performance in an agnostic fashion, resulting in a much bigger number of genes that were reported to be associated with athletic performance. However, the small number of studies in the majority of genes (Table 1) did not allow us to perform a meta-analysis for all these genes that would be properly powered, in statistical terms, restricting us only to the *ACE* and *ACTN3* genes (see also below).

The identification of sports talent is based both on physical and physiological characteristics and the overall performance of athletes in a specific sports discipline. Contemporary studies attempted to correlate genetic loci with athletic performance. Consequently, many genetic testing laboratories offer these services and promise to revolutionize the field of sports. These tests try, through the detection of specific genomic variants, to elucidate the athletic performance potential of individuals, especially the youth. The goal is to contribute to the choice of the appropriate sports career and enable personalized training. Such programs promise to maximize the range of capabilities and minimize the risk of injury.

Herein, from 4228 articles, only 107 (2.53%) were eligible for inclusion in our study, which touched upon 37 different genes and 55 variants (Table 1). From the 37 different genes, for only 2 (5.41%) genes and only for 2 variants, there were enough articles to allow for a proper meta-analysis. Notably, for 27 genomic variants in 22 genes (52.6% of the total number of genes included in our study), there was only a single study that addressed their association with sports performance, while one of these genes, namely the high Fe gene (*HFE*) with the p.C282Y and p.H63D variants, is mostly associated with hemochromatosis (a PubMed search performed in April 2024 with the keywords “HFE” and “Hemochromatosis” revealed 3,174 results). Our data show that only the *ACE* Alu I/D and *ACTN3* p.R577X variants, studied by 29 and 37 articles each, have been adequately studied for their association with elite athletic performance. The next most well-studied variants were the rs4253778 and rs8192678 variants in the Peroxisome proliferator-activated receptor alpha (*PPARA)* and the PARG Coactivator 1 Alpha (*PPARGC1A)* gene, respectively, studied by 9 articles each, that were still not eligible for a proper meta-analysis, and the remaining variants were analyzed in less than 6 studies. Furthermore, the studies mentioned above are characterized by the ambiguity of their outcomes, demonstrating a spectrum of associations between the investigated genetic variant and athletic performance. These associations range from a positive to a weak correlation and, in some instances, no discernible correlation at all.

The complexity of confirming a correlation between a genetic variant and sports performance is further compounded by the heterogeneity in sports discipline and the subjective classification of athletes. In other words, the criteria to assign an athlete to the power or endurance category, according to the skills required by their sport of occupation, are not uniform, as shown in Table 3. The table indicates the sports that fall under the banner of endurance or power sports and their performance level, as defined by each study. Gender and/or nationality were not taken into account. Our data show that despite our careful selection of endurance or power athletes deducted from each article, it is evident that they all come from a variety of different sports. Thus, rowing, cross-country skiing, cross-country skating, polo, handball, duathlon, triathlon, pentathlon, running, swimming, and cycling of medium and long distances were characterized as endurance sports. Conversely, wrestling, boxing, judo, bodybuilding, powerlifting, weightlifting, javelin, high jump, long jump, triple jump, football, kayak, volleyball, ice hockey, and running were characterized as power sports. We opted to follow the athlete and sport classification reported in each of the study included in our meta-analysis and neither to reclassify the athletes’ groups nor to redetermine the type of sport, as this would introduce bias in our analysis. We understand of course that determining an athlete’s performance as elite, sub-elite, etc., cannot be objectively determined, and credible criteria for such classification are currently not established. This is a major limitation of these studies attempting to correlate genomic variants with athletic performance, not to mention the strong influence of epigenetics as well as environmental factors in determining the overall outcome of a world-class competition.

It becomes apparent that the delineation between endurance and power athletes is determined on a study-by-study basis. For example, some studies considered runners up to 400 m as power athletes, while other studies limit this categorization to 100 m sprinters. Moreover, in team sports such as football, not all players can be considered of the same skill, as players’ skills depend on their position in the field, which demands different qualities. Druzhevskaya and coworkers [3] attempted to comprehensively categorize athletes' skills by sport. They revealed that “endurance” skills are also required in sports that belong to the “power” category. Similarly, the definition of the level of athletes differs between studies. However, there is more homogeneity in this classification since many authors comply with the categorization in terms of level, as defined by Druzhevskaya and coworkers [3] and this was the reason to select this classification for the purpose of this meta-analysis.

Upon analyzing the quality of the associations between genes and athletic performance as reported in case–control studies, our meta-analysis revealed inconsistencies in the findings related to *ACE* Alu I/D and *ACTN3* p.R577X variants. It was noted that a positive correlation was found in a relatively small studies with well-defined groups of athletes, while a negative correlation was found in larger studies with a more diverse athletes’ population. This fact, coupled with the varied methodology pursued in each study, and confounders such as the sample size, the inclusion of different sports leading to phenotypic heterogeneity, and ethnic diversity makes it exceedingly challenging to draw firm conclusions regarding the association of these two variants with elite sport performance. For example, considering the aspect of ethnicity, Kenya is known to be home to some of the best runners in the world. If a genomic variant is found to be correlated with endurance in Kenyan runners, it is imperative to confirm the same association in runners of different ethnic backgrounds.

Several sports teams consider the results of these genetic tests seriously when making direct coaching recommendations, as indicated in the literature [2, 9, 14]. While genome-wide association studies (GWAS) have identified possible associations between genes and athletic performance at the study population level, the possible association of each variant at an individual’s level is less consistent. Genetic testing alone cannot conclusively confirm or rule out an individual’s athletic performance [1].

On the other hand, implementing genetic testing for sports performance carries significant ethical considerations. For instance, children who aspire to become world-class athletes can have several negative effects, such as depression and psychological problems, if the genotype supposedly associated with their preferred sport is not detected. Conversely, the results may falsely reassure them that they will become top athletes. Considering these concerns, the governance of sports genomics should be stringently regulated by ethics review committees, aligning with the principles outlined in the Declaration of Helsinki (World Medical Association, 2008), while any genome-guided recommendations for athletic performance should abide to regulatory approval and not simply interpretation of the findings which could be subjective. Moreover, general public access to such tests should be revised considerably. As such, it would be reasonable for all reported genetic associations to remain in the investigational sphere before being allowed to be released to the market until two critical conditions are met: (a) there is a biologically plausible and well-supported molecular mechanism by which the variant could impact athletic performance and (b) replication of the positive association between the variant and athletic performance in several independent studies in different populations. This is essential to mitigate the risk of false positive results that would mislead the interested individuals.

Overall, genomic markers cannot per se predict athletic performance for talent identification due to the multifaceted nature of athletic performance, which is influenced not only by our genetic background but also, and significantly, by various environmental factors. Regarding the genetic background, it is critical to determine other variants associated with resistance to injuries or the ability to recover from them. Furthermore, it is important to consider the association of these variants with pathogens, as has been found for the *ACE* gene. On the other hand, the difficulty in predicting, let alone simulating, the interplay of environmental factors with one’s genetic profile to contribute to one's final sports performance is unquestionable.

The data from the present study underline that sports genetics is a promising discipline that warrants additional research that includes larger and more homogeneous and well-defined athlete groups from several ethnicities. Without definitive data, the commercial availability of genetic testing for athletic performance to the general public poses significant ethical and safety concerns. Therefore, stringent regulatory oversight by health and legislative bodies is imperative to safeguard the general public against premature application of such genetic testing services.

## Supplementary Information

Supplementary material 1.

## Data Availability

Data used in this article are available by the authors upon request.
