# Supplementary material for: Meta-analysis of genomic variants in power and endurance sports to decode the impact of genomics on athletic performance and success
Source: Hum Genomics. 2024 May 17;18:47. doi: 10.1186/s40246-024-00621-9 (PMC11102131; doi:10.1186/s40246-024-00621-9)
Supplement: Supplementary file 1 — Supplementary material 1. [file 40246_2024_621_MOESM1_ESM.pdf]

Supplementary Information

Meta-analysis of genomic variants in power and endurance sports to decode the impact of genomics on athletic performance and success

Aikaterini Psatha [1], Zeina N. Al-Mahayri [1,2], Christina Mitropoulou [2,4], George P. Patrinos [1,3,4,5,*]

[1] University of Patras, School of Health Sciences, Department of Pharmacy, Laboratory of Pharmacogenomics and Individualized Therapy, Patras, Greece

[2] The Golden Helix Foundation, London, UK

[3] Erasmus University Medical Center, Faculty of Medicine and Health Sciences, Department of Pathology, Clinical Bioinformatics Unit, Rotterdam, the Netherlands

[4] United Arab Emirates University, College of Medicine and Health Sciences, Department of Genetics and Genomics, Al-Ain, Abu Dhabi, UAE

[5] United Arab Emirates University, Zayed Center for Health Sciences, Al-Ain, Abu Dhabi, UAE

Supplementary Figure 1. Forest plot to correlate the *ACE* I allele with the athletic performance of **endurance** athletes versus non-athlete control individuals. The comparison includes *ACE* II + *ACE* ID versus *ACE* DD genotypes.

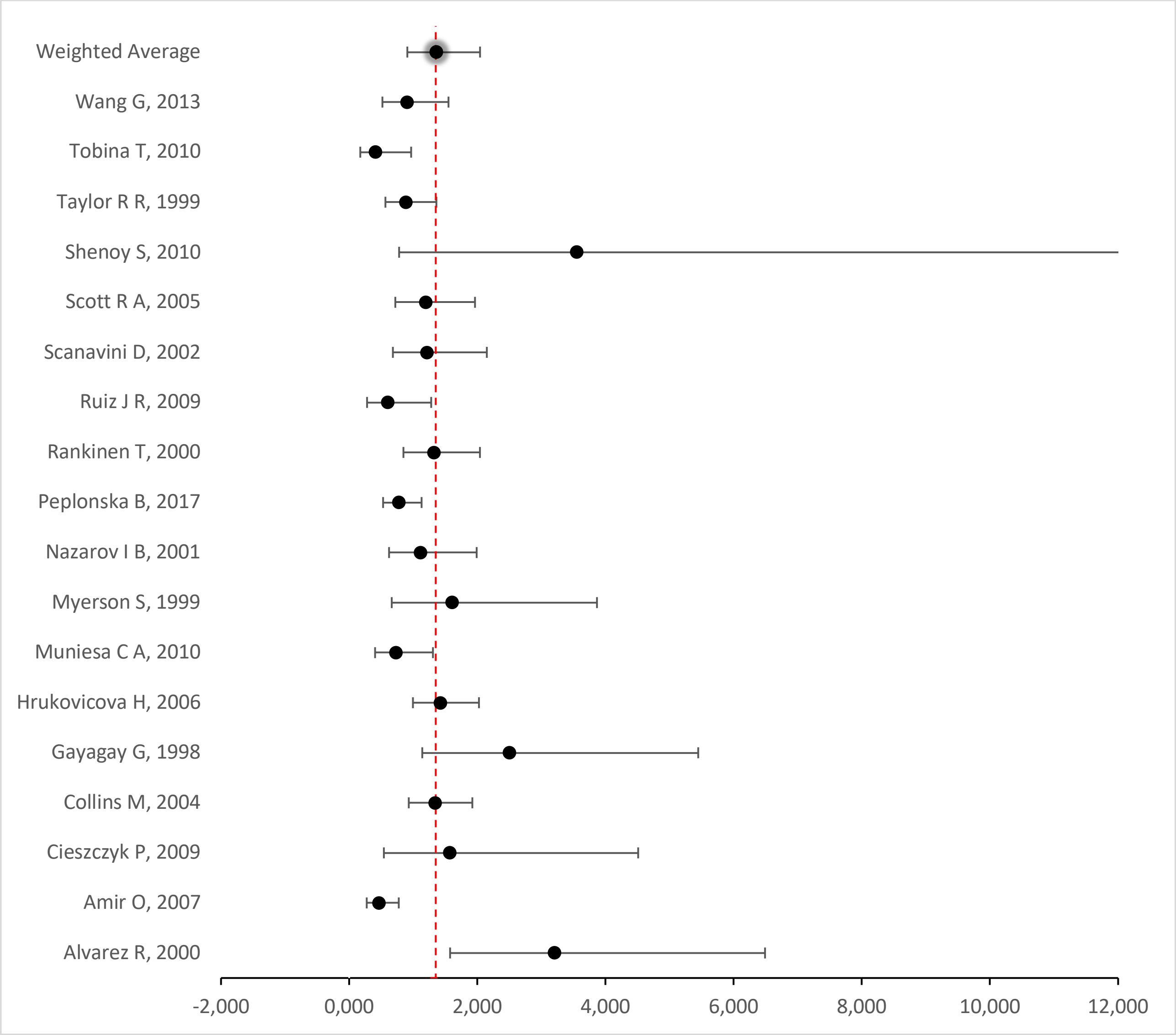

Supplementary Figure 2. Forest plot to correlate the *ACE* I allele with the athletic performance of **power** athletes vs non-athlete control individuals. The comparison includes *ACE* II + *ACE* ID versus *ACE* DD genotypes.

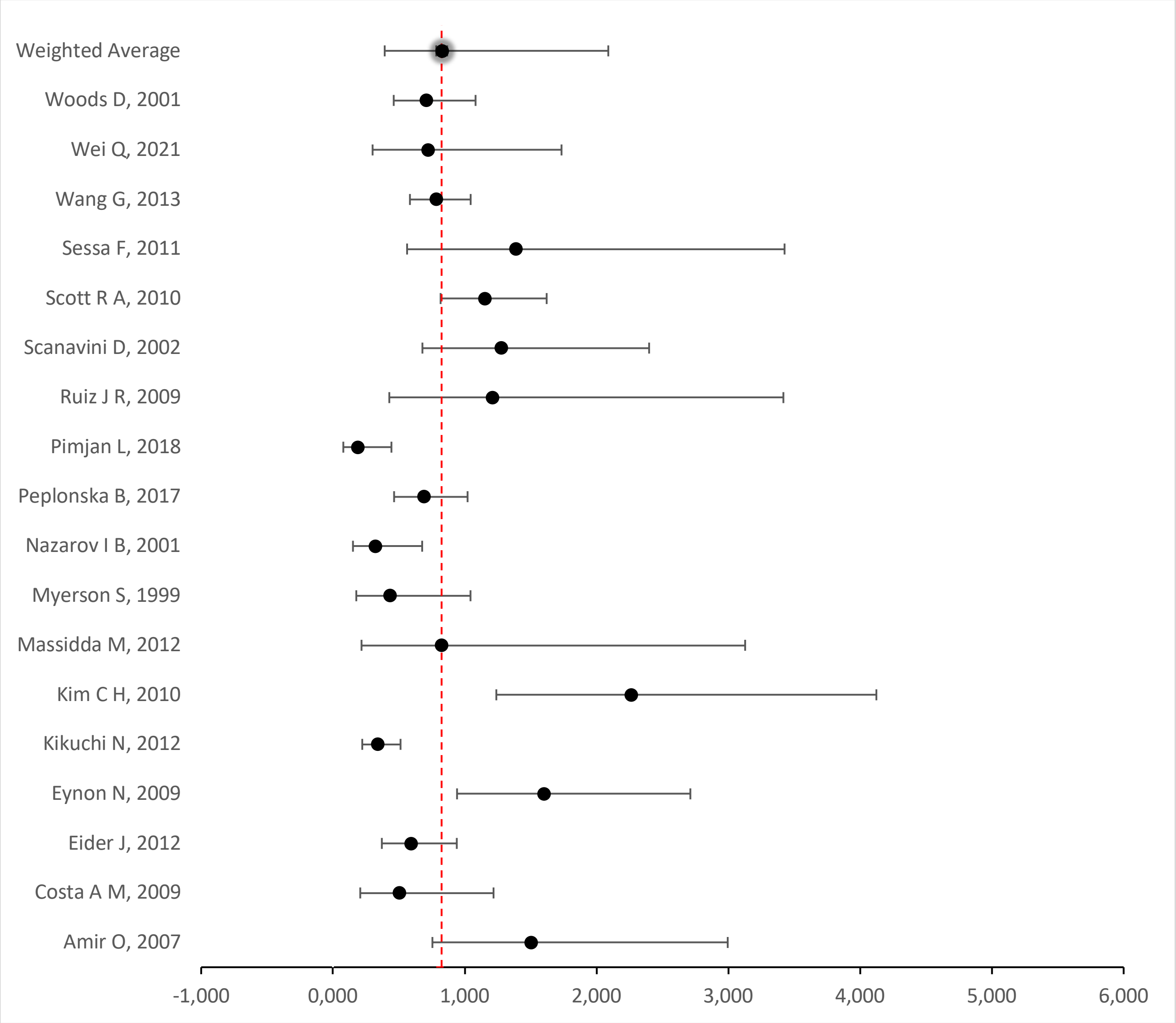

Supplementary Figure 3. Forest plot to correlate the *ACE* I allele with the athletic performance of **endurance and power** athletes vs non-athlete control individuals. The comparison includes *ACE* II + *ACE* ID versus *ACE* DD genotypes.

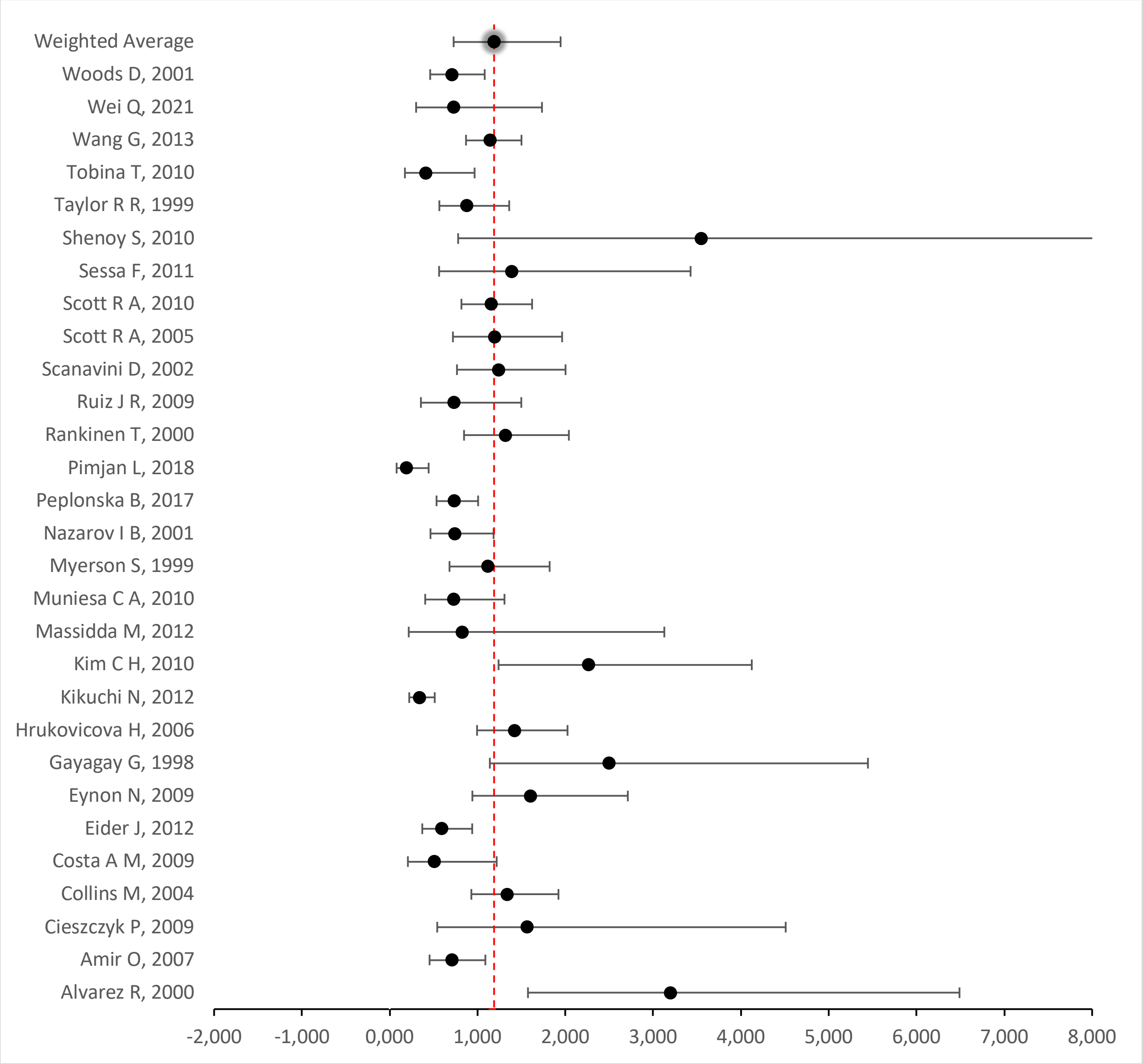

Supplementary Figure 4. Forest plot to correlate the *ACE* I allele with the athletic performance of **endurance** athletes vs non-athlete control individuals. The comparison includes *ACE* II versus *ACE* ID + *ACE* DD genotypes.

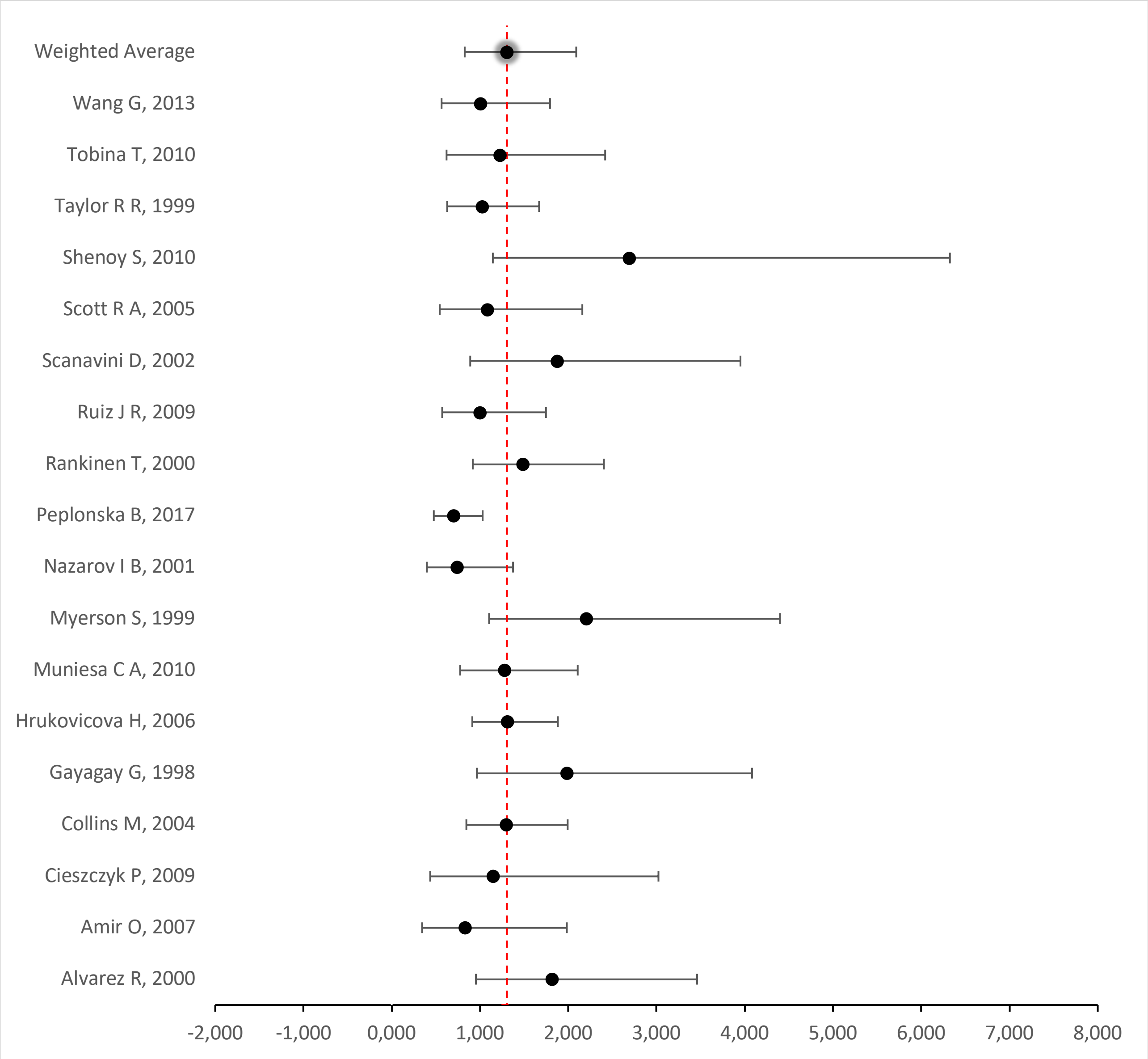

Supplementary Figure 5. Forest plot to correlate the *ACE* I allele with the athletic performance of **power** athletes versus non-athlete control individuals. The comparison includes *ACE* II versus *ACE* ID + *ACE* DD genotypes.

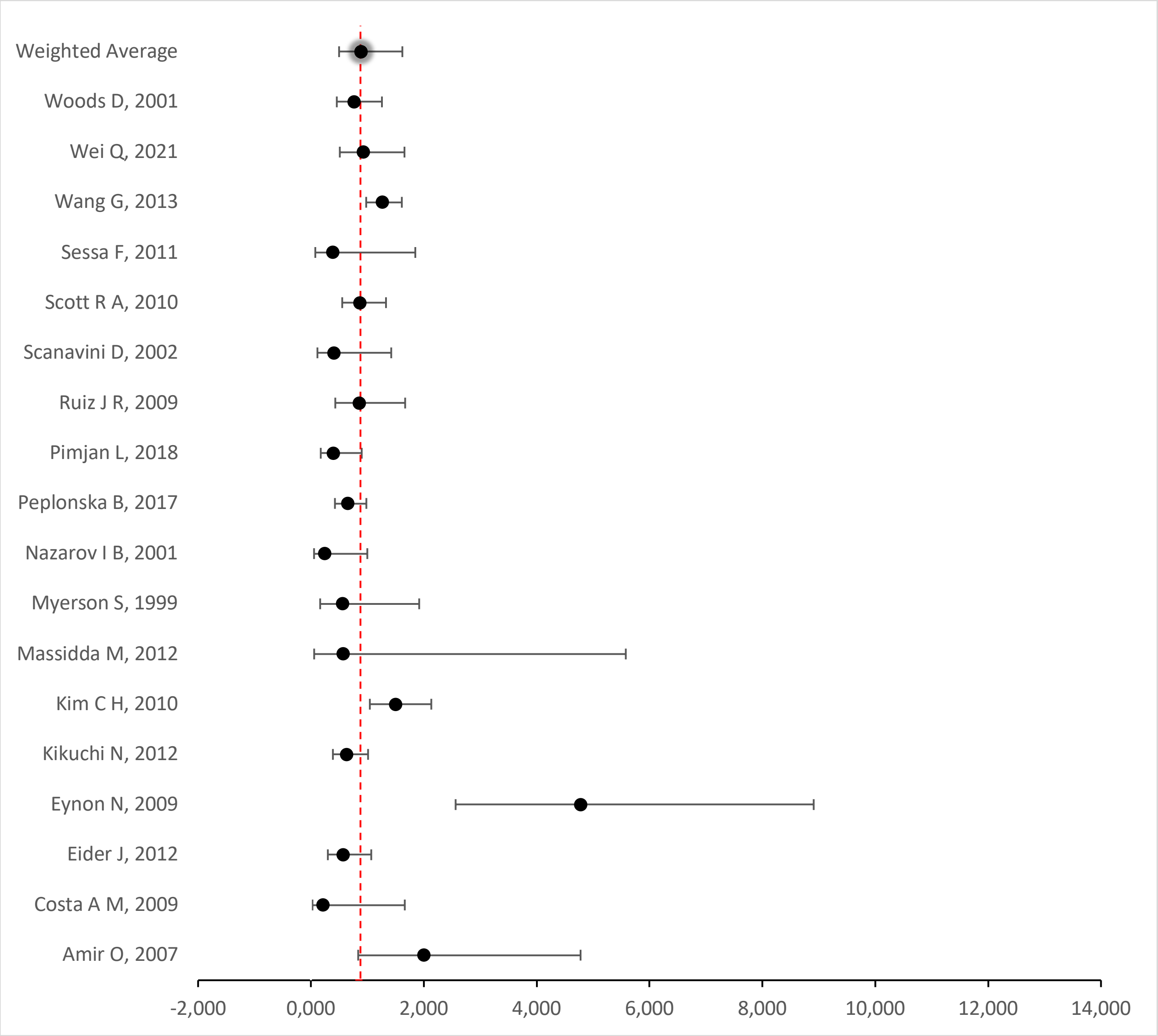

Supplementary Figure 6. Forest plot to correlate the *ACE* I allele with the athletic performance of **endurance and power** athletes versus non-athlete control individuals. The comparison includes *ACE* II versus *ACE* ID + *ACE* DD genotypes.

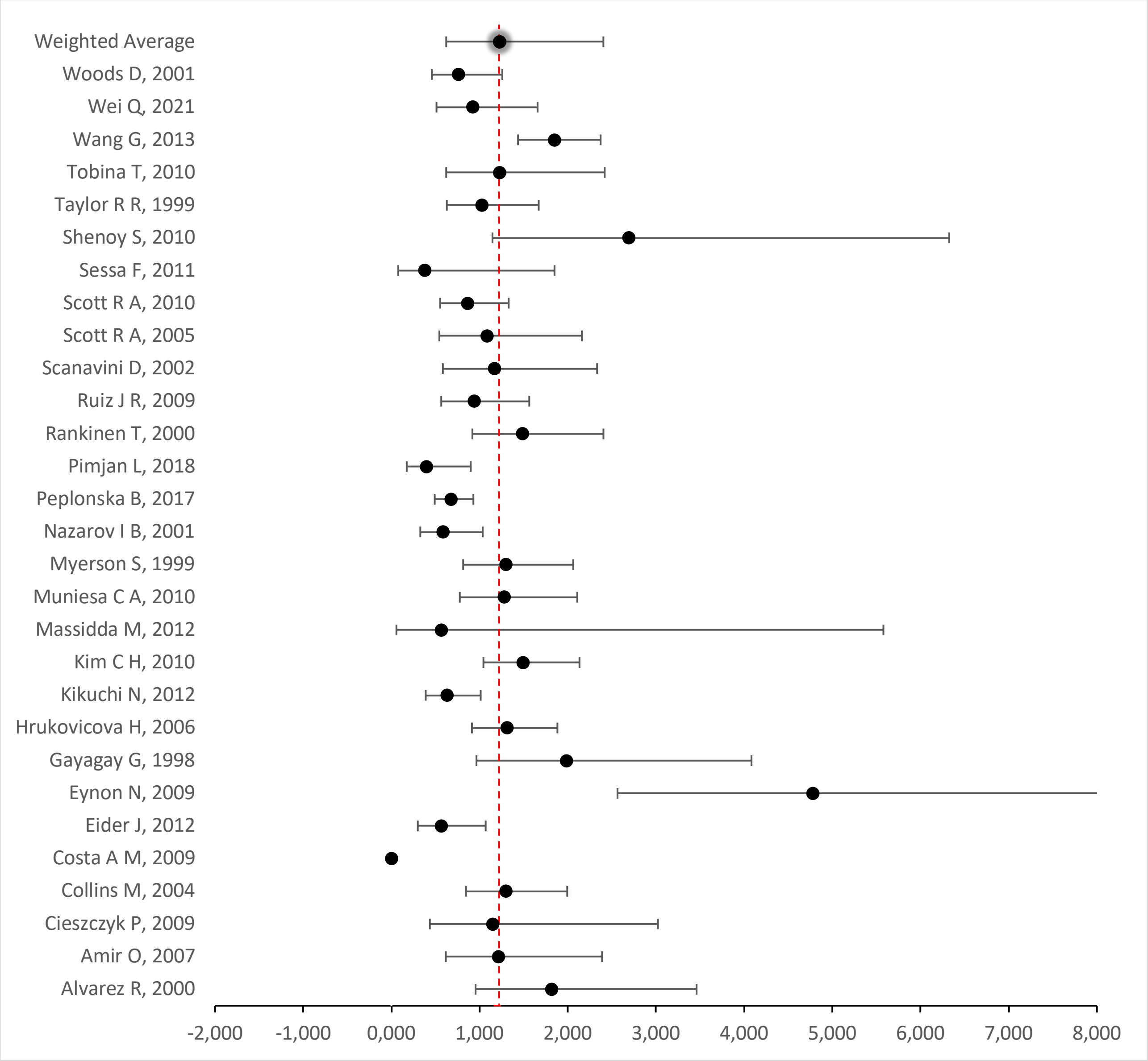

Supplementary Figure 7. Forest plot to correlate the *ACTN3* p.R577X allele with the athletic performance of **endurance** athletes vs non-athlete control individuals. The comparison includes *ACTN3* p.577RR + *ACTN3* p.577RX versus *ACTN3* p.577XX genotypes.

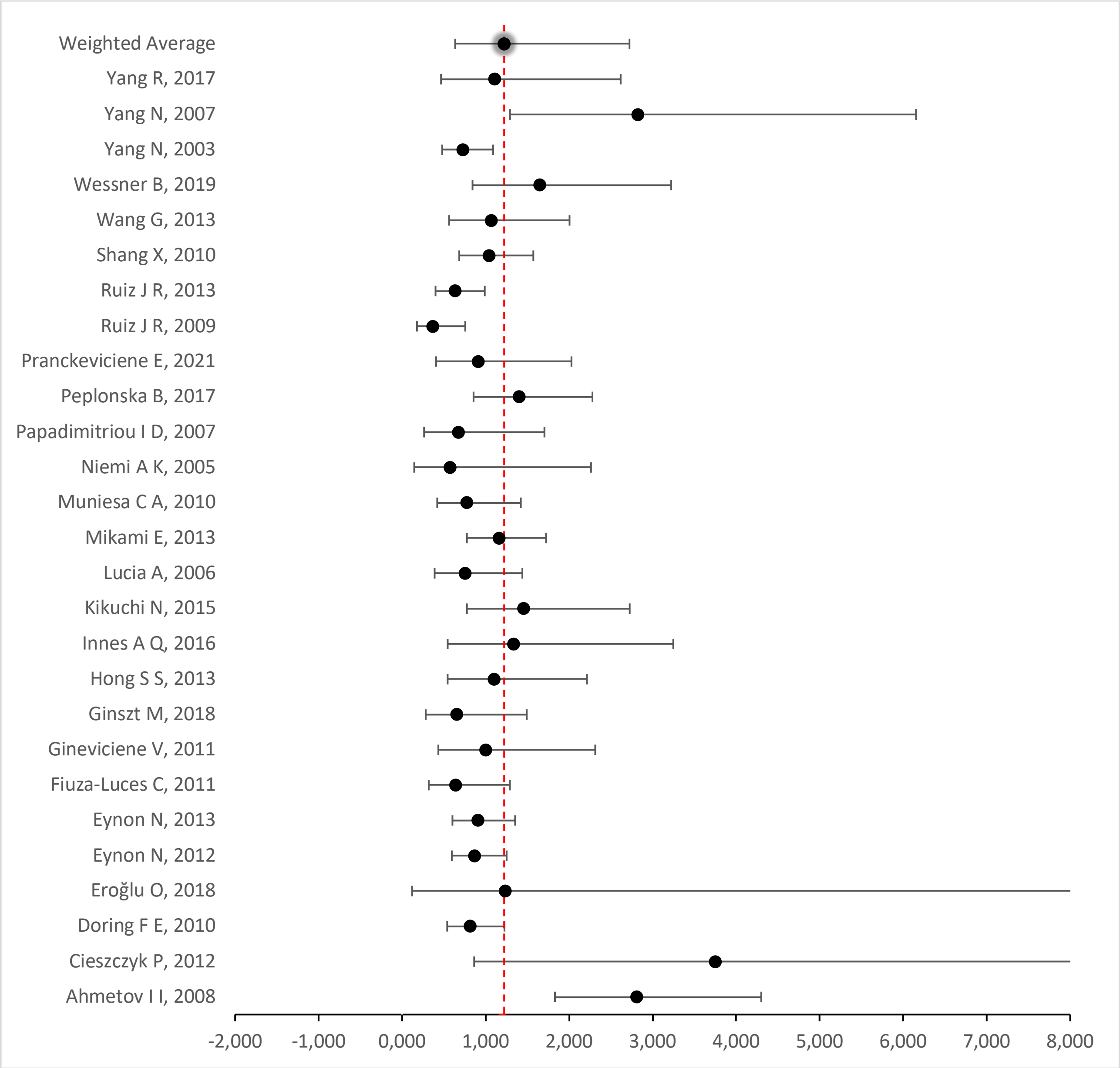

Supplementary Figure 8. Forest plot to correlate the *ACTN3* p.R577X allele with the athletic performance of **power** athletes versus non-athlete control individuals. The comparison includes *ACTN3* p.577RR + *ACTN3* p.577RX versus *ACTN3* p.577XX genotypes.

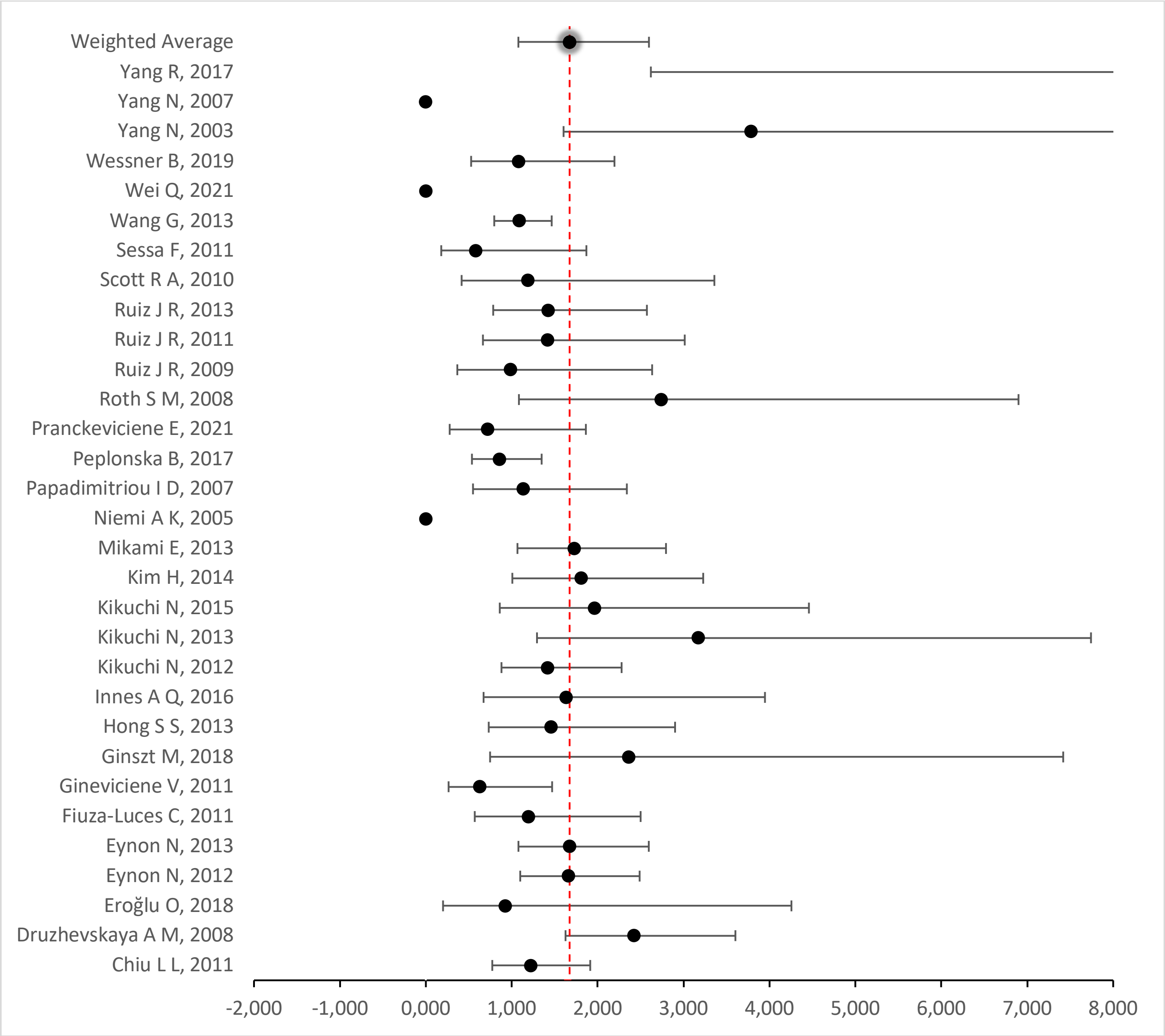

Supplementary Figure 9. Forest plot to correlate the *ACTN3* p.R577X allele with the athletic performance of **endurance and power** athletes versus non-athlete control individuals. The comparison includes *ACTN3* p.577RR + *ACTN3* p.577RX versus *ACTN3* p.577XX genotypes.

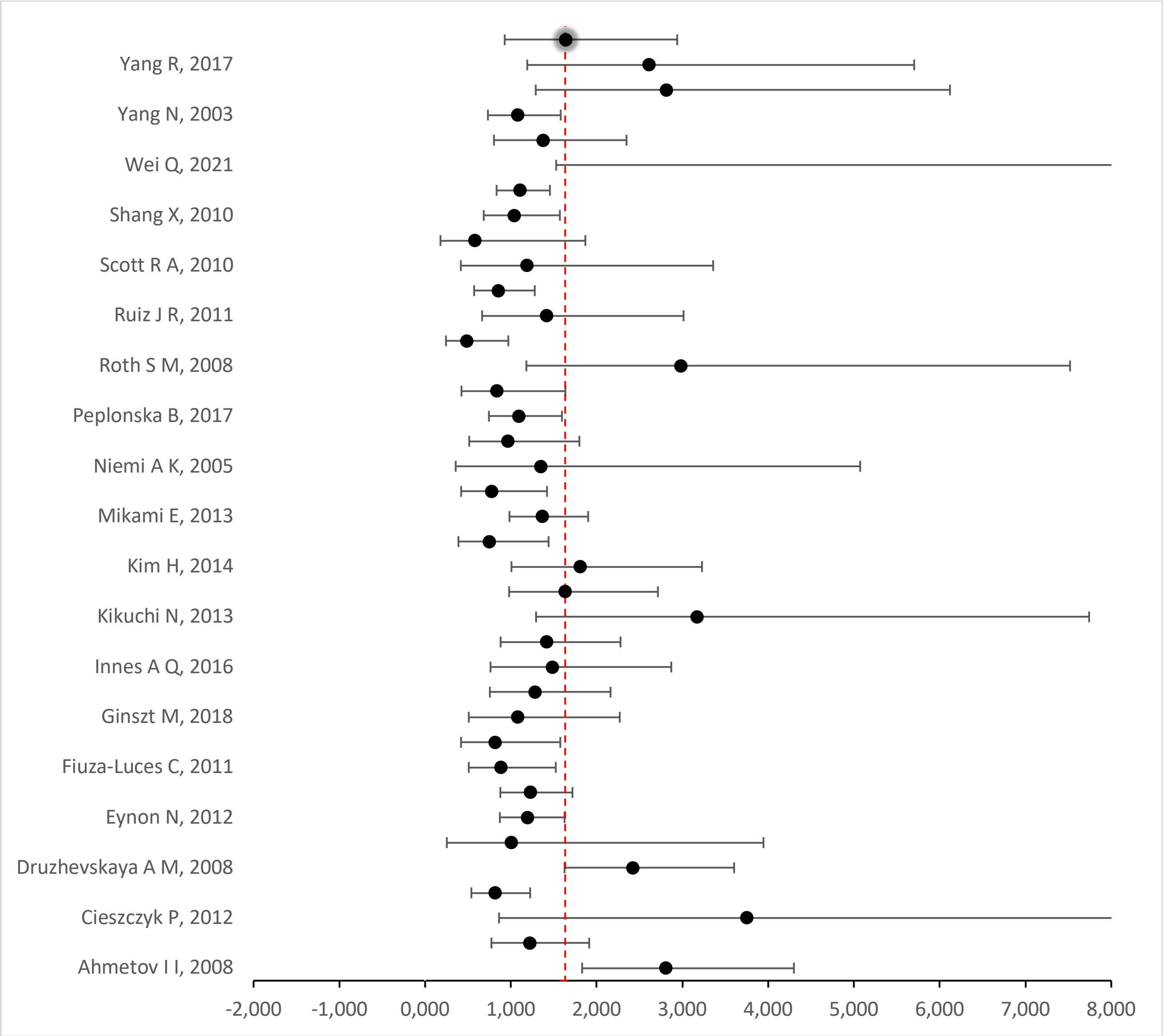

Supplementary Figure 10. Forest plot to correlate the *ACTN3* p.R577X allele with the athletic performance of **endurance** athletes versus non-athlete control individuals. The comparison includes *ACTN3* p.577RR vs *ACTN3* p.577RX + *ACTN3* p.577XX genotypes.

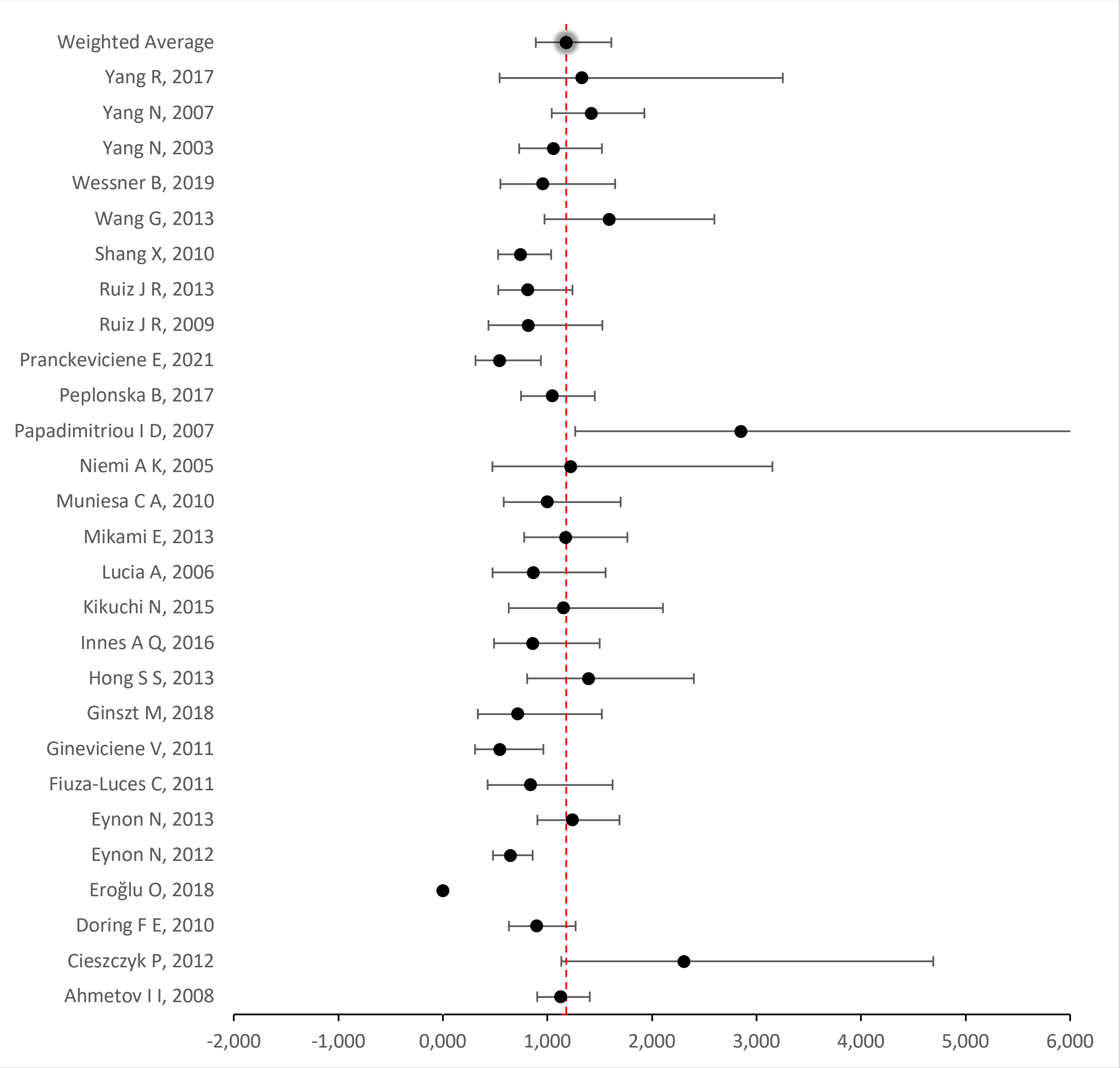

Supplementary Figure 11. Forest plot to correlate the *ACTN3* p.R577X allele with the athletic performance of **power** athletes versus non-athlete control individuals. The comparison includes *ACTN3* p.577RR vs *ACTN3* p.577RX + *ACTN3* p.577XX genotypes.

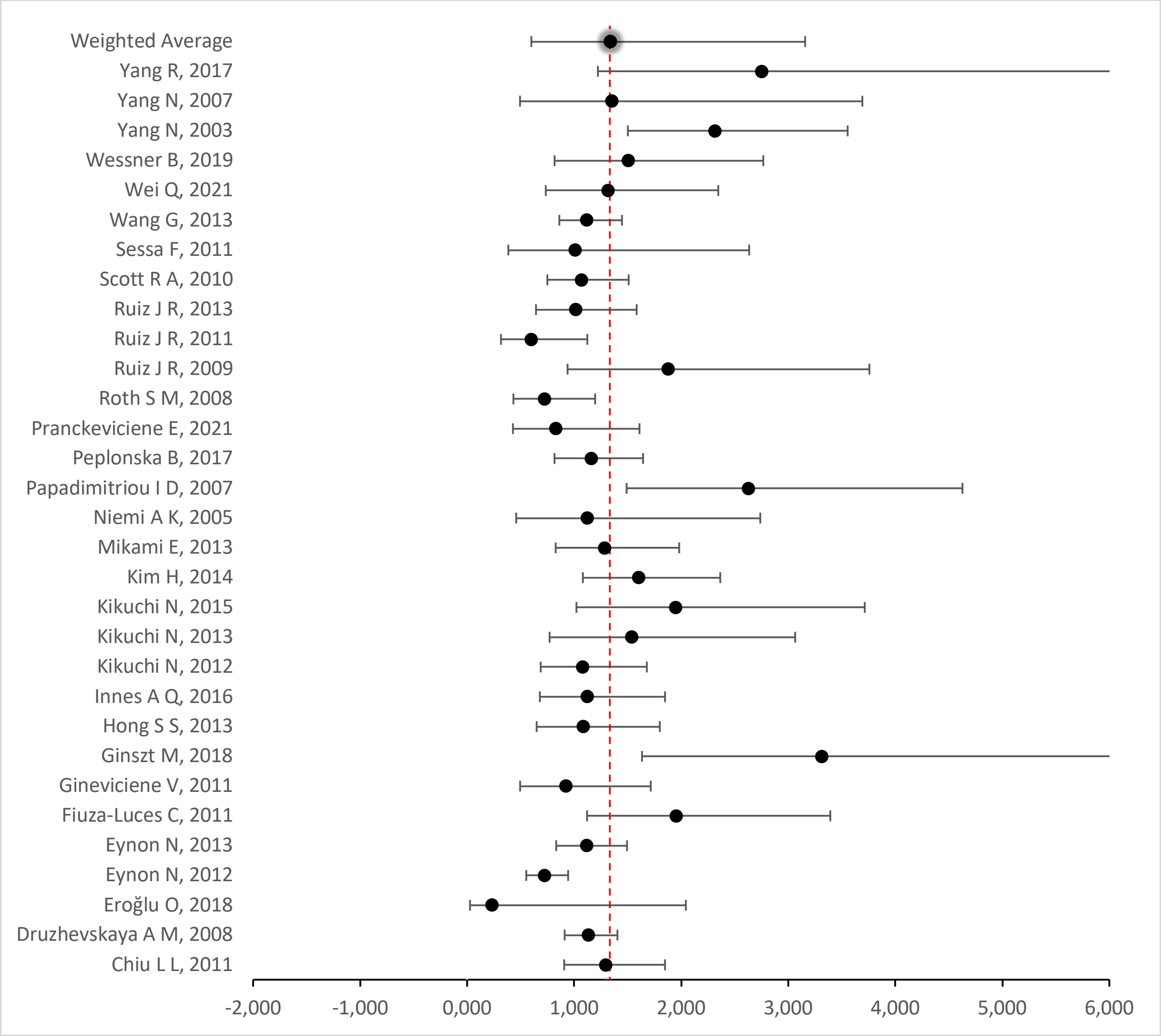

Supplementary Figure 12. Forest plot to correlate the *ACTN3* p.R577X allele with the athletic performance of **endurance and power** athletes versus non-athlete control individuals. The comparison includes *ACTN3* p.577RR vs *ACTN3* p.577RX + *ACTN3* p.577XX genotypes.

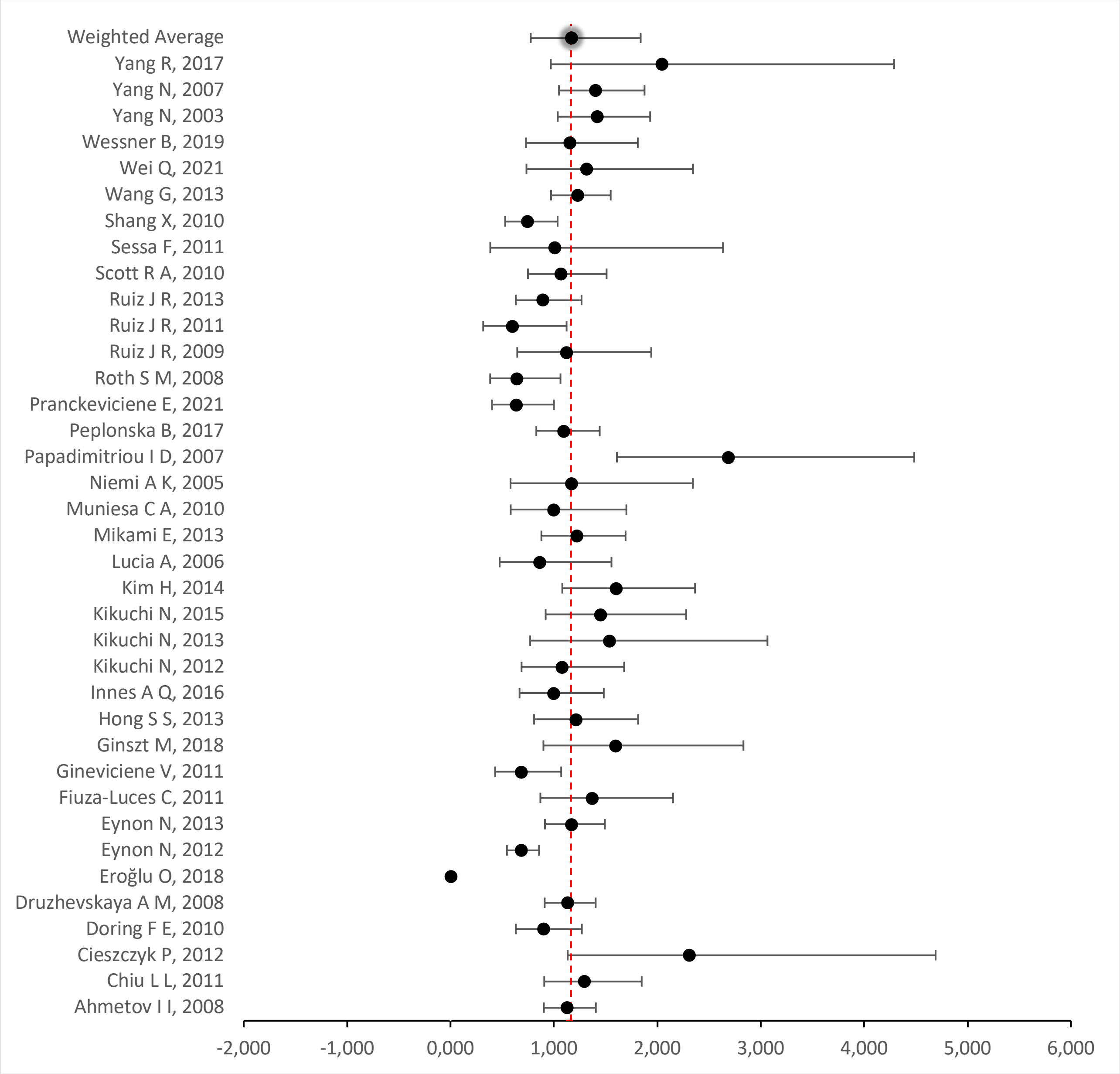

Supplementary Figure 13. Histogram indicating the differences in the *ACE* I and *ACE* D allelic frequencies between **endurance athletes** (in dark blue) and **controls** (in orange; p=0.3435).

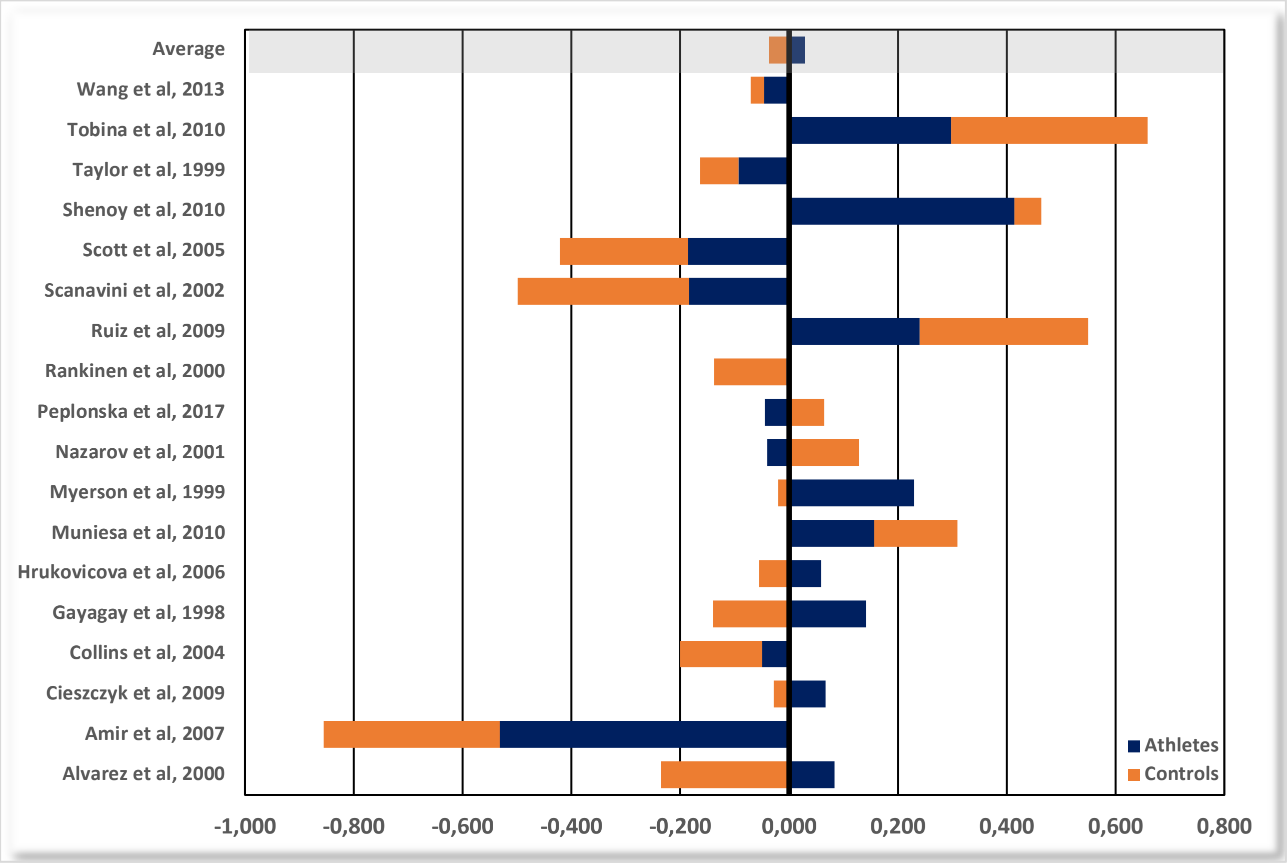

Supplementary Figure 14. Histogram indicating the differences in the *ACE* I and *ACE* D allelic frequencies between **power athletes** (in dark blue) and **controls** (in orange; p=0.1768).

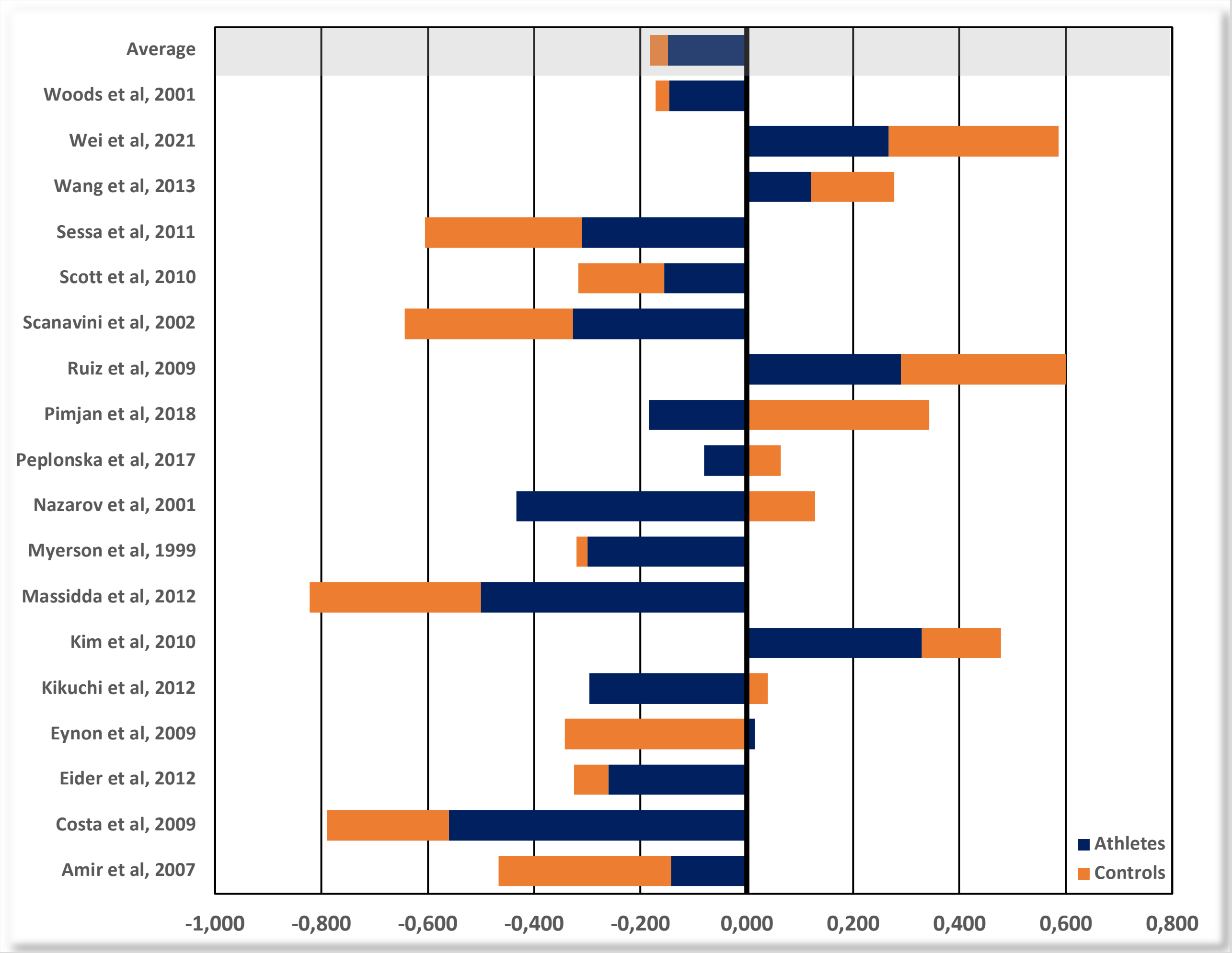

Supplementary Figure 15. Histogram indicating the differences in the *ACTN3* p.577R and *ACTN3* p.577X allelic frequencies between **endurance athletes** (in dark blue) and **controls** (in orange; p=0.8634).

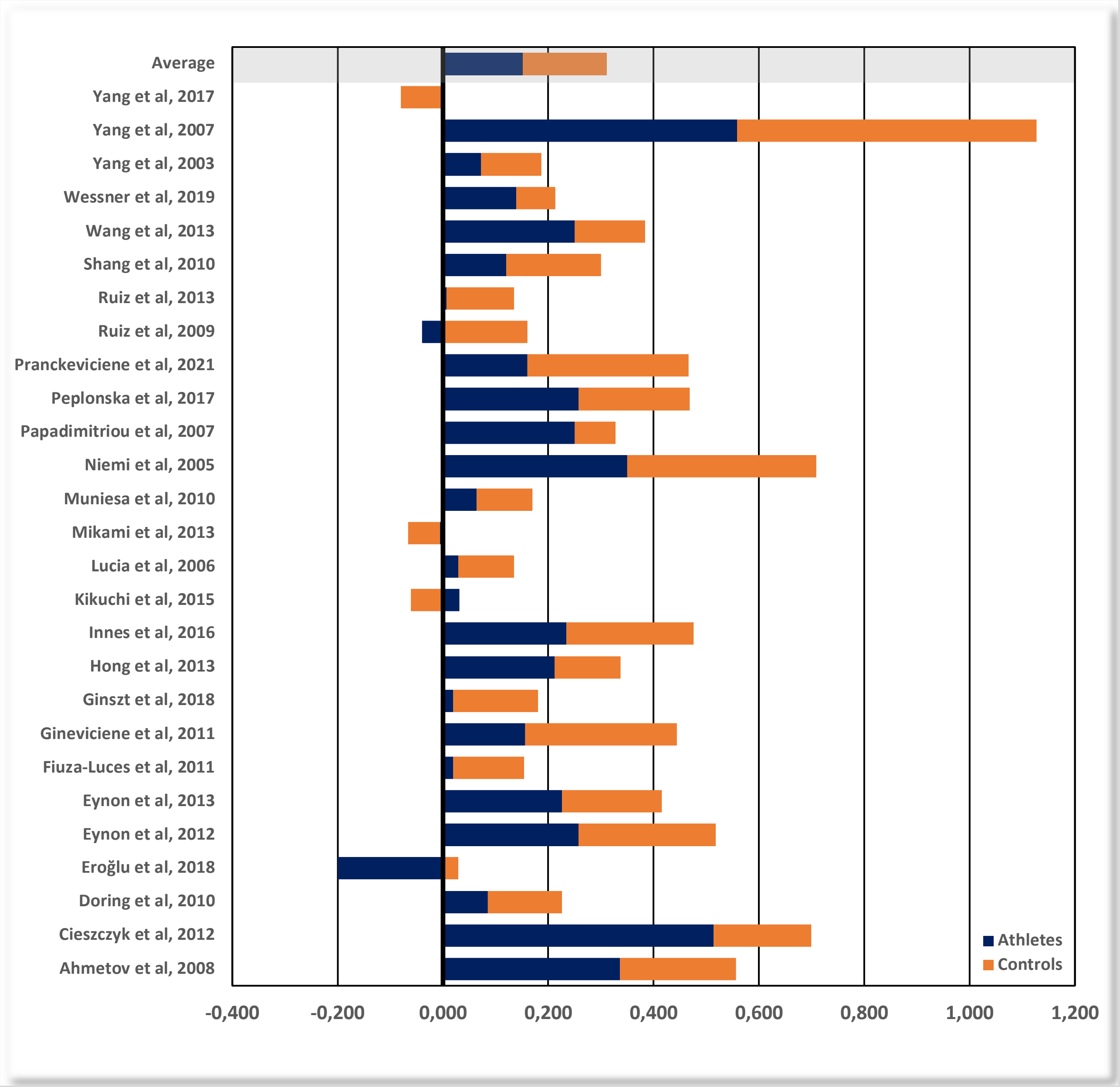

Supplementary Figure 16. Histogram indicating the differences in the *ACTN3* p.577R and *ACTN3* p.577X allelic frequencies between **power athletes** (in dark blue) and **controls** (in orange; p=0.052).

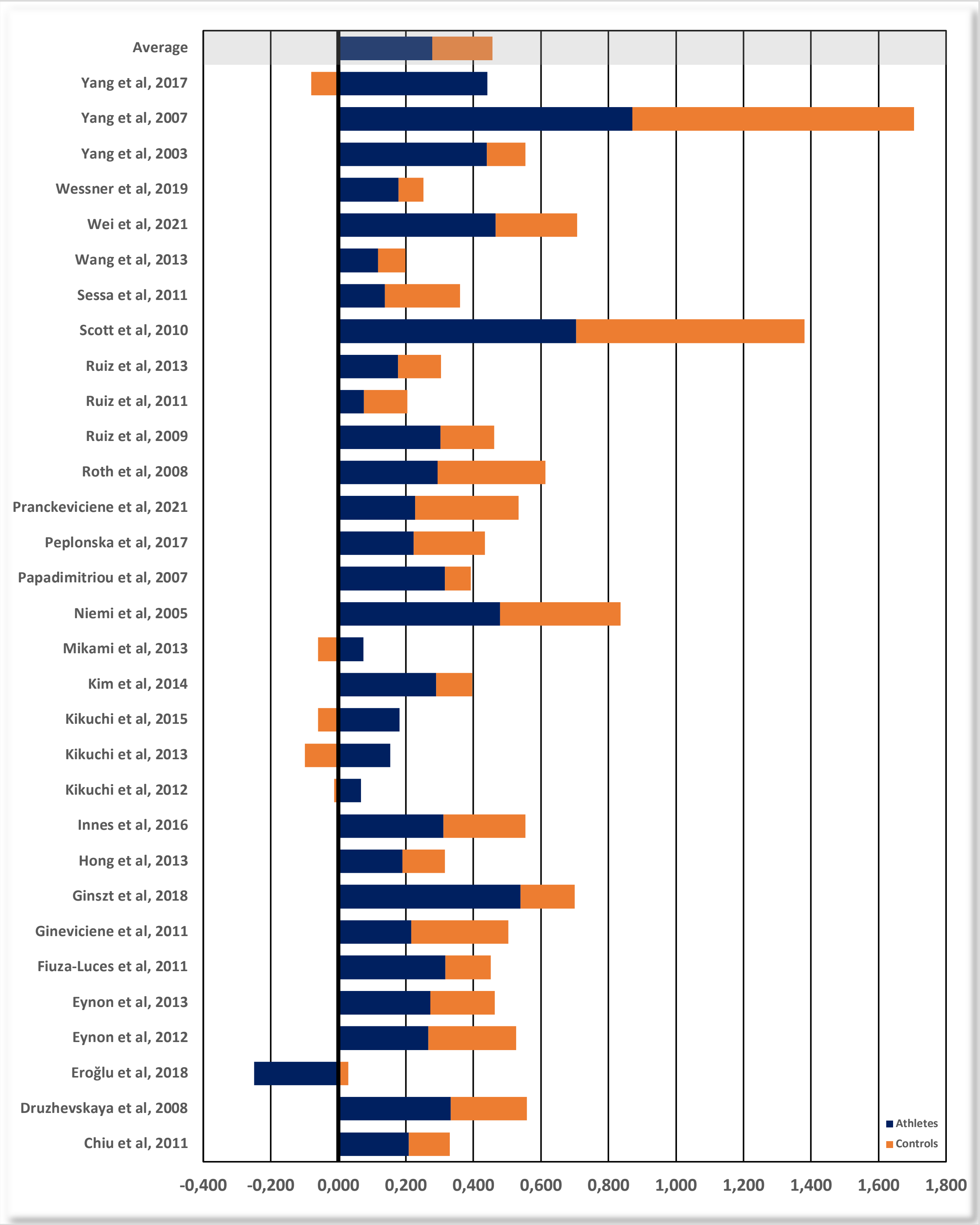

Supplementary Table 1. Summary of the articles that were included in the present meta-analysis.

No	Study (PubMed ID or DOI)	Disciplines	Sport definition in articles	Gene(s)
1	Ahmetov et al., 2008 (18718976)	Endurance	Biathlon, cross-country skiing, race walking, road cycling, rowing, swimming 800 – 2500 m, triathlon	*ACTN3*
2	Alvarez et al., 2000 (10879452)	Endurance	Cycling, long distance running, handball	*ACE*
3	Amir et al., 2007 (17631516)	Endurance	Marathon	*ACE*
		Power	Sprint 100 – 200 m	
4	Chiu et al., 2011 (21472630)	Power	Swimming ≤ 400 m	*ACTN3*
5	Cieszczyk et al., 2009 (19455482)	Endurance	Rowing	*ACE*
6	Cieszczyk et al., 2012 (https://doi.org/10.1016/j.jesf.2012.04.003)	Endurance	Rowing	*ACTN3*
7	Collins et al., 2004 (10797114)	Endurance	Triathlon	*ACE*
8	Costa et al., 2009 (19458960)	Power	Short distance swimming 50 – 200 m	*ACE*
9	Doring et al., 2010 (20845221)	Endurance	Cross-country skiing, bi- and triathlon, cycling, running, rowing	*ACTN3*
10	Druzhevskaya et al., 2008 (18470530)	Power	Biathlon, cross-country skiing, race walking, road cycling, rowing, swimming 800 – 2500 m, triathlon	*ACTN3*
11	Eider et al., 2012 (http://dx.doi.org/10.1016/j.scispo.2012.11.005)	Power	Running 100 – 400 m, powerlifting, weightlifting, throwing, jumping	*ACE*
12	Eroğlu et al., 2018 (29729690)	Endurance	Long distance running	*ACTN3*

		Power	Short distance running	*ACTN3*
13	Eynon et al., 2009 (20013558)	Power	Running 100 – 200 m	*ACE*
14	Eynon et al., 2012 (22916217)	Endurance	Running 5000 m - marathon, road cycling, rowing, swimming 800 – 1500 m, cross-country skiing 15 – 50 km, triathlon, skating ≥ 5000 m, walkers, swimming >200 m, duathlon, water polo	*ACTN3*
		Power	Weightlifting, sprinting ≤ 200 m, jumping, volleyball, ice hockey, skating ≤ 1000 m, boxing, wrestling, swimming ≤ 200 m, weightlifting, figure skating, shot putting, heavy event, taekwondo	
15	Eynon et al., 2013 (23522773)	Endurance	Running 5000 m-marathon, road cycling, rowing, swimming 800 – 1500 m, cross-country skiing 15 – 50 km, triathlon, skating ≥ 5000 m, walkers, swimming > 200 m, duathlon, water polo	*ACTN3*
		Power	Weightlifting, sprinting ≤ 200 m, jumping, volleyball, ice hockey, skating ≤ 1000 m, boxing, wrestling, swimming ≤ 200 m, weightlifting, figure skating, shot putting, heavy event, taekwondo	
16	Fiuza-Luces et al., 2011 (21407828)	Endurance	Road cycling	*ACTN3*
		Power	Jumping, sprint	
17	Gayagay et al., 1998 (9737775)	Endurance	Rowing	*ACE*
18	Gineviciene et al., 2011 (21956137)	Endurance	Long distance running, middle distance running	*ACTN3*
19	Ginszt et al., 2018 (29401200)	Endurance	Lead climbing	*ACTN3*
		Power	Bouldering	*ACTN3*

20	Hong et al., 2013 (https://doi.org/10.1007/s13258-013-0111-7)	Endurance	Long distance cycling, rowing, swimming ⩾ 400 m, track athletes ⩾ 5000 m, cross-country skiing	*ACTN3*
		Power	Track athletes ⩽800 m, swimming ⩽200 m, judo, short-distance track cycling, speed skating	
21	Hruskovicova et al., 2006 (16998460)	Endurance	Marathon, half-marathon, inline skating	*ACE*
22	Innes, 2016 (http://hdl.handle.net/1893/23287)	Endurance	Long distance cycling, modern pentathlon, orienteering, running, skiing, race walking	*ACTN3*
		Power	Weightlifting, bodybuilding, gymnastics, short distance swimming, short distance running	
23	Kikuchi et al., 2012 (22996021)	Power	Wrestling	*ACE, ACTN3*
24	Kikuchi et al., 2013 (22868563)	Power	Wrestling	*ACTN3*
25	Kikuchi et al., 2015 (26324221)	Endurance	Middle distance running 800 – 1500 m, long distance running ≥ 3000 m	*ACTN3*
		Power	Short sprint 100 – 200 m, long sprint 400 m, jumping, throwing, decathlon, heptathlon	
26	Kim et al., 2010 (20029740)	Power	Discus, hammer, javelin, high, long and triple jump, pole vault, running 100 – 200 m, weightlifting	*ACE*
27	Kim et al., 2014 (25671201)	Power	Sprint ≤ 400 m, speed skating ≤ 1500 m, swimming ≤ 200 m	*ACTN3*
		Power	Weightlifting	
28	Lucia et al., 2006 (16612741)	Endurance	Middle distance running ~1500 m, long-distance running, marathon, cycling	*ACTN3*
29	Massidda et al., 2012 (22648472)	Power	Running 100 – 400 m	*ACE*

30	Mikami et al., 2013 (23868678)	Endurance	Long distance running ≥ 5000 m, middle-distance running 800 – 3000 m, race walking	*ACTN3*
		Power	Sprint ≤ 400 m, jumping, throwing, decathlon	
31	Muniesa et al., 2010 (18801770)	Endurance	Endurance running, cycling, rowing	*ACE, ACTN3*
32	Myerson et al., 1999 (10517757)	Endurance	Running ≥ 5000 m	*ACE*
		Power	Running ≤ 200 m	
33	Nazarov et al., 2001 (11781693)	Endurance	Long distance running	*ACE*
		Power	Short distance running	
34	Niemi et al., 2005 (15886711)	Endurance	Running 800 m – marathon	*ACTN3*
		Power	Sprint	
35	Papadimitriou et al., 2007 (17879893)	Endurance	Long distance running 3000 m - marathon, middle distance running 800 – 1500 m, triathlon, race walking	*ACTN3*
		Power	Sprint 100 – 400 m, jumping, throwing, decathlon	
36	Peplonska et al., 2017 (27140937)	Endurance	Running 3000 m marathon, cross-country skiing, speed skating 3000 – 10000 m, swimming 400 – 1500 m, modern pentathlon, orienteering, biathlon, mountain bike, road cycling, triathlon, rowing, canoeing, race walking	*ACE, ACTN3*
		Power	Sprint 100 – 400 m, swimming 50 – 100 m, speed skating 100 – 500 m, track cycling 200 – 1000 m)	
37	Pimjan et al., 2018 (https://doi.org/10.48048/wjst.2018.3525)	Power	Weightlifting	*ACE*
38	Pranckeviciene et al., 2021 (34356082)	Endurance	Very long, long and medium distance athletes: skiers, road cyclists, bi-athletes, long-distance	*ACTN3*

			runners, modern pentathletes, swimmers, rowers	
		Power	Sprinters and other power athletes with predominantly anaerobic energy production: sprinters, jumpers, throwers	
39	Rankinen et al., 2000 (10797114)	Endurance	Cross-country skiing, biathlon, long-distance running, middle distance running, road cycling	*ACE*
40	Roth et al., 2008 (18043716)	Power	Bodybuilding, powerlifting	*ACTN3*
41	Ruiz et al., 2009 (20044471)	Endurance	Distance running, road cycling	*ACE, ACTN3*
		Power	Jumping, sprint	
42	Ruiz et al., 2011 (20561285)	Power	Volleyball	*ACTN3*
43	Ruiz et al., 2013 (23317015)	Endurance	Long distance running 5000 m – marathon, road cycling, rowing	*ACTN3*
		Power	Volleyball, jumping, sprint	
44	Scanavini et al., 2002 (12357325)	Endurance	Road cyclists, track and field running, cross-country skiing	*ACE*
		Power	Flat-water kayak	
45	Scott et al., 2005 (15950509)	Endurance	Running 3000 m – marathon	*ACE*
46	Scott et al., 2010 (20010124)	Power	Running < 400 m, jumping, throwing	*ACE, ACTN3*
47	Sessa et al., 2011 (21254885)	Power	Sprint, short distance swimming, volleyball	*ACE, ACTN3*
48	Shang et al., 2010 (20936592)	Endurance	Rowing, running ≥ 5000 m, marathon, long distance cycling, swimming ≥ 400 m	*ACTN3*
49	Shenoy et al., 2010 (22375202)	Endurance	Triathlon	*ACE*
50	Taylor et al., 1999 (10484574)	Endurance	Hockey, cycling, skiing, track and field, swimming, rowing, gymnastics, other	*ACE*

51	Tobina et al., 2010 (20574690)	Endurance	Long distance running > 5000 m	*ACE*
52	Wang et al., 2013 (23190598)	Power	Swimming short and middle distance ≤ 400 m	*ACE, ACTN3*
		Endurance	Swimming long distance > 400 m	
		Power	Swimming short distance ≤ 100 m	
53	Wei et al., 2021 (33602343)	Power	Soccer	*ACE, ACTN3*
54	Wessner et al., 2019 (https://doi.org/10.34045/SSEM/2016/21)	Endurance	Middle and long distance running, road cycling, triathlon, biathlon	*ACTN3*
		Power	Sprint, jumping, throwing, weightlifting	
55	Woods et al., 2001 (11354635)	Power	Swimming ≤ 400m	*ACE*
56	Yang et al., 2003 (12879365)	Endurance	Long distance cycling, rowing, swimming ≥ 400 m, track ≥ 5000 m, cross-country skiing	*ACTN3*
		Power	Track ≤ 800 m, swimming ≤ 200 m, judo, short distance track cycling, speed skating	
57	Yang et al., 2007 (17986906)	Endurance	Running 3000 m – marathon	*ACTN3*
		Power	Running ≤ 400 m, 110 m hurdle, jumping	
58	Yang et al., 2017 (27442335)	Endurance	Running 5000 m – marathon	*ACTN3*
		Power	100 m and 200 m sprint, jumping, shot putting, discus throwing, javelin throwing, weightlifting, 500 m track sprint cycling	
